# Supplementary material for: Translating Guidelines into Practice: A Multicentre Audit of the Implementation of ERC Survivorship and Follow-Up Recommendations After Cardiac Arrest
Source: J Clin Med. 2025 Dec 25;15(1):174. doi: 10.3390/jcm15010174 (PMC12786956; doi:10.3390/jcm15010174)
Supplement: Supplementary file 1 [file jcm-15-00174-s001.zip › Supplementary Material S3.pdf]

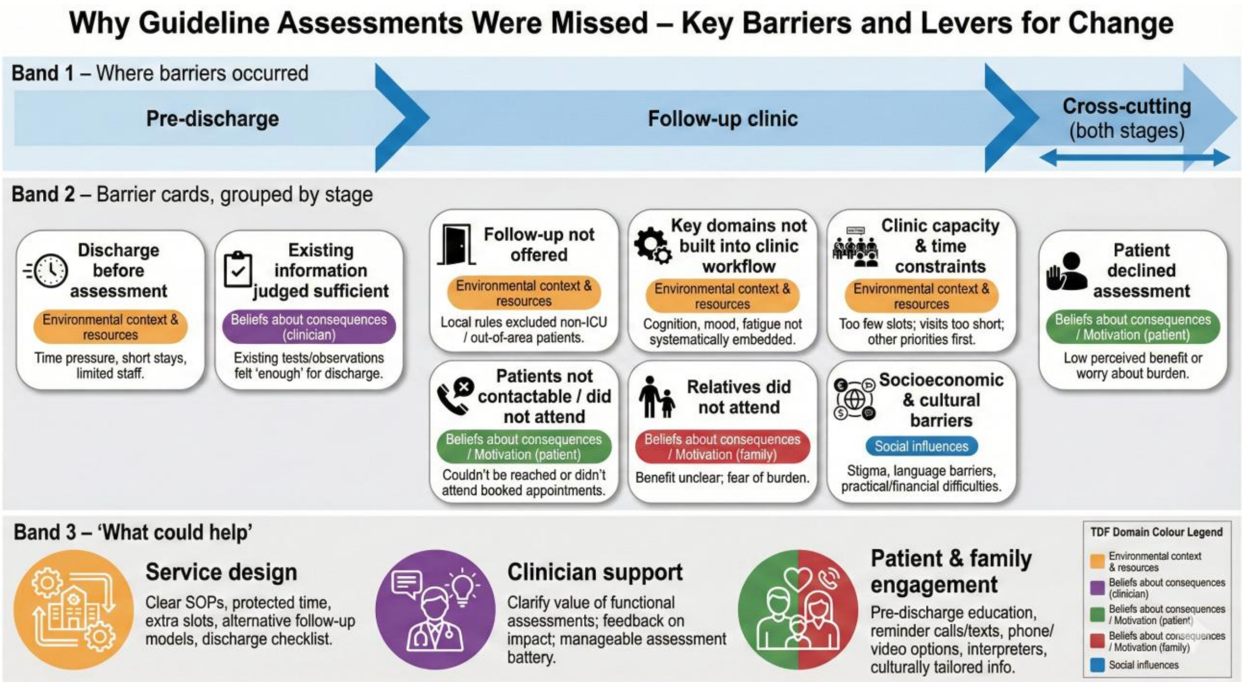

Supplement Figure S1. Infographic showing where and why guideline-recommended assessments were not delivered across the OHCA pathway, grouping barriers by stage and TDF domain and linking them to service-, clinician- and patient/family-level solutions (colours denote TDF domains).
