# Supplementary material for: Translating Guidelines into Practice: A Multicentre Audit of the Implementation of ERC Survivorship and Follow-Up Recommendations After Cardiac Arrest
Source: J Clin Med. 2025 Dec 25;15(1):174. doi: 10.3390/jcm15010174 (PMC12786956; doi:10.3390/jcm15010174)
Supplement: Supplementary file 1 [file jcm-15-00174-s001.zip › Supplementary Material S1.pdf]

Supplementary Material S1

Table S1. – List of cognitive assessments/mood screen questionnaires in use at the time of the Audit in the 4 hospital Trusts.

Standardized assessment of cognition	'Functional cognition'	Assessment of fatigue	Mood Screen
**MoCA**<br>**MoCA-Telephone or audio-visual**<br>**FreeCog**<br>**Tele-Cog**<br>**ACE-III**	Multiple Errands Task (MET)<br>Kitchen-based Functional Assessment<br>PADLs Assessment<br>METs + PADLs<br>MET + Kitchen Tasks<br>Kitchen Tasks + PADLs	MFIS short version<br>MFIS long version<br>VAF (pre-post)	HADS<br>PHQ-9 and GAD-7

MoCA – Montreal Cognitive Assessment; FreeCog – Free Cognitive Assessment Tool; ACE-III – Addenbrooke's Cognitive Examination, version III; MoCA-Telephone – MoCA adapted for telephone use; Tele-Cog – Remote/telehealth-delivered cognitive assessment; MET – Multiple Errands Test; PADLs – Performance-based Activities of Daily Living; MFIS – Modified Fatigue Impact Scale; VAF – Visual Analogue Fatigue scale; HADS – Hospital Anxiety and Depression Scale; PHQ-9 – Patient Health Questionnaire-9; GAD-7 – Generalized Anxiety Disorder-7
